# Health and Social Vulnerabilities Among Unstably Housed and Homeless Young Adults During the COVID-19 Pandemic

**DOI:** 10.1177/15248399231217447

**Published:** 2023-12-15

**Authors:** Jessica A. Heerde, Barbara J. McMorris, Janna R. Gewirtz O’Brien, Jennifer A. Bailey, John W. Toumbourou

**Affiliations:** 1 The University of Melbourne, Melbourne, Victoria, Australia; 2 Murdoch Children’s Research Institute, Melbourne, Victoria, Australia; 3 University of Minnesota, Minneapolis, MN, USA; 4 University of Washington, Seattle, WA, USA; 5 Deakin University, Melbourne, Victoria, Australia

**Keywords:** health equity, health disparities, health promotion, social determinants of health

## Abstract

The role of housing as a social driver of health is well-established, with stable housing being an important factor in reducing health inequities. During developmentally critical periods such as young adulthood, unstable housing and related social marginalization have profound effects on development and later health, social, and economic wellbeing. This exploratory study analyzed data from a population-based, longitudinal sample of young adults (average age 31 years) from Washington State (*n* = 755) to compare health and economic impacts of the early days of the COVID-19 pandemic, with a focus on housing status. Descriptive results suggest the pandemic exposed underlying vulnerabilities for young adults experiencing homelessness and housing instability, with an overall widening of inequities related to financial difficulties and increased risk for poor mental health and social isolation. Findings suggest that these vulnerabilities are magnified in the context of public health crises and strengthen the case for population-based studies investigating potential modifiable causes of housing instability to inform prevention and early intervention at the earliest possible point in a young person’s development. Studies examining the severity of COVID-related hardships on young adult health and social outcomes are vital for establishing an evidence base for strategic policy action that seeks to prevent a rebound in young adult homelessness and housing instability post-pandemic. These studies would bolster both emergency preparedness responses that account for the unique needs of vulnerable populations and upstream population-level prevention approaches beginning long before the imminent risk for housing instability develops.

Housing status and health are inextricably linked (Marmot, 2022). Social marginalization at developmentally critical periods such as young adulthood has a negative impact on development, health, and socioeconomic wellbeing (Scales et al., 2016). Homelessness and housing instability, a form of social marginalization, continue to rise globally, with profound effects on preventable morbidity (Heerde & Patton, 2020). Safe and affordable housing provides individuals with stability, agency, and a foundation for health and socioeconomic security (Shaw, 2004).

Despite concerns about their increased susceptibility to the virus, socially marginalized groups were largely excluded from the initial COVID-19 response planning (Gewirtz O’Brien et al., 2021). As such, the pandemic highlighted the absence of coordinated policies addressing the provision of health and social care to those experiencing marginalization, in general and particularly during public health crises. This prompted urgent local and national policy action to mitigate the socioeconomic impact of the pandemic, particularly for those who might be most vulnerable (e.g., eviction moratoriums, investments in unemployment) (Boston University, 2023). Emerging evidence suggests that the pandemic and its associated socioeconomic impacts exacerbated health inequities (Abrams & Szefler, 2020; Wang & Tang, 2020).

The purpose of the current study was to use longitudinal data to compare health and economic impacts of the early days of the COVID-19 pandemic between young adults experiencing homelessness and housing insecurity and their stably housed peers.

## Methods

This descriptive study analyzed quantitative and brief qualitative data collected from American young adults participating in the International Youth Development Study (IYDS). The IYDS is an ongoing, cross-national longitudinal study following state-representative samples from Washington State, United States, and Victoria, Australia.

Full details on study methods were previously published (McMorris et al., 2007). Briefly, original sampling and recruitment used a probability-proportionate to grade-level size sampling procedure with standardized methodologies in both states. Eligible Washington State students in fifth, seventh, and ninth grades (*N* = 3,856) were approached to participate. Of these, a state-representative sample of 2,885 students (74.8%) consented to and took part in the 2002 survey. Data from the Washington State young adult cohort who were seventh graders in 2002 were analyzed here (*n* = 755). Retention rates for this cohort have remained high: 86% at age 25 (in 2014), 88% at age 29 (2018), and 83% at age 31 (2020).

Human subjects approval was obtained from the University of Washington Institutional Review Board. In 2020, the online survey took 50–60 minutes to complete. Participants were reimbursed USD $40. Over 95% of participants completed the survey between April and August 2020, mostly in April (55%) and May (24%), coinciding with the beginning of lockdowns in the United States during the early months of the pandemic.

### Measures

The IYDS survey uses self-report measures of a range of health and social behaviors adapted from the Communities That Care youth survey. The current study focused on measures of housing status and the impact of the pandemic.

Housing status was assessed using two items self-reported at average ages of 25, 29, and 31 years: “In the past year, have you been homeless (i.e., not had a regular place to live)?” and “Which of the following best describes where you currently live?” In line with international definitions of homelessness (Amore et al., 2011), homelessness was scored as 1 (otherwise 0) if respondents replied yes to the first question or responded that they were, for example, currently “staying with friends temporarily” or living in a “refuge/temporary accommodation,” or “hotel/motel/caravan,” reflecting the forms of homelessness young adults experience (Heerde et al., 2020). Four housing status groups were defined: (1) never been homeless/unstably housed, (2) homeless/unstably housed in young adulthood prior to age 31, (3) newly homeless/unstably housed at age 31, and (4) chronically homeless/unstably housed at all three adult time points.

The impact of the COVID-19 Pandemic was assessed using items adapted from the work by Temple et al. (2020) asking about effects on finances, employment, and mental health and whether participants and their family members had been tested for COVID-19. To assess the effects of social distancing, participants also responded to the following open-ended question,

Many people have been required to stay at home and limit contact with others during the COVID-19 pandemic. In your own life, you may have noticed good things, bad things, or perhaps no changes at all related to this “social distancing.” Please briefly describe whether and how social distancing is affecting you during the COVID-19 (coronavirus) pandemic.

Demographic data included participant-reported age, sex (male/female), employment status, racial identity, zip code, whether they had ever been married, if they identified as being a parent, health insurance status, and with whom they currently live.

### Analyses

Stata/SE version 15.1 was used to conduct descriptive analyses of survey data. Small subsample sizes limited capacity to test for statistical differences between the four housing status groups. Open-ended qualitative responses were examined using a categorical narrative approach in which responses were read multiple times by the lead authors (JAH and BJM) who identified patterns and themes within the participants’ responses. A list of framing codes and categories were generated for each theme. The authors discussed the emergent categories and themes and each other’s interpretations of the data. Exemplar quotes only from those who had ever experienced housing instability were used to illustrate findings from the descriptive comparison of survey responses across the four housing status groups.

## Results

Table 1 shows sample demographics. Approximately 9.7% of young adults reported unstable housing in the past year (prior to 2020), 1.3% reported chronic unstable housing during adulthood, and 1.2% reported unstable housing for the first time in 2020. The impacts of the pandemic were distributed unequally across these groups (see Table 2), with chronically and newly unstably housed individuals reporting the worst outcomes and formerly unstably housed individuals reporting worse outcomes than those who never experienced unstable housing. For example, rates of financial strain, food insecurity, and stress, anxiety, and depression were very high among newly and chronically unhoused individuals.

**Table 1:** Characteristics of the Study Population

Sample characteristics	Full sample (*n* = 775), % (*n*)
Demographics	
Age (years), mean (*SD*)	31.3 (0.44)
Female	55.9 (422)
Genderqueer/non-binary/gender non-conforming	0.7 (5)
Transgender	0.1 (1)
Have you ever been married (yes; %, *N*)	53.1 (401)
Race and ethnicity	
Asian	5.7 (43)
Native American	7.6 (57)
Black	4.4 (33)
Spanish, Hispanic, Mexican	11.1 (84)
Hawaiian, Pacific Islander	2.6 (20)
White	74.3 (561)
Other	0.5 (4)
Household members	
*Who do you currently live with?(select all that apply)*	
Alone	9.5 (72)
Mom	9.0 (68)
Dad	5.8 (39)
Step-parent or your partners’ parent	0.8 (6)
Siblings	5.6 (42)
Other relatives	1.7 (13)
Boyfriend, girlfriend, or spouse	60.0 (453)
Boyfriend, girlfriend, or spouse’s family	1.8 (14)
With house-mates or friends	7.4 (56)
Your children	39.1 (295)
Other adults not biologically related to you	4.6 (35)
Other children not biologically related to you	2.6 (20)
Anyone else	4.6 (35)
Parenting	
*Are you the parent of a child?(select all that apply)*	
No	47.4 (358)
Yes, biological parent	50.9 (384)
Yes, adoptive parent	1.1 (8)
Yes, foster parent	0.4 (3)
Yes, step-parent	5.2 (39)
Current sources of income/finances	
*What are your sources of income? (select all that apply)* Paid employment	83.7 (632)
Parental support	4.4 (33)
Spousal support	9.4 (71)
Government support	10.9 (82)
Other	10.5 (79)
Do you have savings? (Yes)	72.6 (548)
Health	
Health insurance (Yes)	90.6 (684)

**Table 2:** Differential Impacts of Early COVID-19 Pandemic Lockdowns

Financial, social and health outcomes	Chronic unstable housing prior to age 31 and at age 31 (*n* = 10) (%)	Newly unstable housed at age 31 (*n* = 9) (%)	Unstably housed prior to age 31 (*n* = 73) (%)	Never unstably housed (*n* = 660) (%)
Finances & employment				
*Financial situation before the COVID pandemic*:				
Living comfortably	0.0	0.0	26.0	39.8
Doing alright	33.3	33.3	46.6	44.2
Just getting by or finding it quite or very difficult	66.7	66.7	27.4	15.9
*Because of the COVID-19 pandemic*:				
Lost job or had hours reduced	55.6	44.4	38.9	33.3
Household member lost job or had hours reduced	62.5	62.5	47.0	40.9
Had trouble buying adequate food sometimes or often	88.9	44.4	20.7	7.9
Had trouble paying the rent because of money problems sometimes or often	66.7	55.6	23.3	9.3
Had trouble paying electric, heating, cooling, or other household bills sometime or often.	55.6	44.4	20.5	8.7
Social isolation & mental health:				
*Because of the COVID-19 pandemic*:				
Feel lonely sometimes or often	66.7	88.9	46.6	37.4
Feel stressed, anxious, or depressed sometimes or often	88.9	100.0	57.5	59.1
Health				
Been tested for COVID-19	0.0	0.0	5.5	7.4
Household member tested for COVID-19	0.0	12.5	15.2	9.6

*Note.* COVID-19 = coronavirus disease 2019.

Open-ended responses were very consistent with the quantitative data. Although losing a job or having hours reduced were reported by most of the sample, those participants who were chronically or newly unstably housed were particularly impacted. For example, a newly unstably housed participant wrote, “*I can’t work my main job as a bartender currently so less income*.” Difficulty affording food, rent, and utilities also was a common theme among chronically or newly unstably housed individuals. As one chronically unstably housed participant wrote, “*I am spending most of my time at home, with the occasional trip to the food bank or grocery store*.”

The unequal impacts of the pandemic were not limited to financial circumstances but also extended to mental and physical health. Greater numbers of participants who were chronically or newly unstably housed reported feeling stressed, anxious, or depressed “sometimes” or “often,” compared to their peers. As this newly unstably housed participant wrote, “*Social Distancing has made anxiety and depression more apparent due to being stuck inside and having to wear masks*.” And as this chronically unstably housed participant offered, “*. . . it has severely impacted my Wife’s mental health [isolation] to the point that she attempted suicide*.”

Another emergent and recurrent theme in the qualitative data, closely related to mental health, was loneliness and isolation. For example, “*. . . I normally go to doctor’s appointments and [mental health] treatment, now those things are done over the phone, so I do feel pretty isolated*” was volunteered by a chronically unstably housed participant. Relatedly, this participant who reported being chronically unstably housed offered, “*My birthday has been canceled. My family members not living here are lonely and stressed and my brother needs hands on support but can’t get it.*” For this formerly unstably housed participant, loneliness and isolation was aggravated by stay-at-home orders recalling: “*Struggling to support work/life balance when work from home is becoming part of the norm.*”

With respect to physical health, testing for COVID-19 was not widespread during the early months of the pandemic; however, it was essentially nonexistent for those who reported being unstably housed (refer to Table 2). Two participants who were unstably housed during adulthood but prior to 2020 wrote about differences in the health care experience, “*I am currently 8 months pregnant. Because of Covid-19, I cannot have anyone go to doctors’ appointments with me. I’m only allowed one support person with me when I give birth*” and “*I’ve gained weight from being home and bored*.”

## Discussion

The role of stable housing as a social determinant of health is well-established. Our findings expand upon the existing literature suggesting that the COVID-19 pandemic magnified underlying health and socioeconomic vulnerabilities for young adults experiencing housing instability (Abrams & Szefler, 2020; Gewirtz O’Brien et al., 2021; Wang & Tang, 2020). This was despite unprecedented federal, state, and local pandemic-driven housing and social investments, including investment in temporary and emergency accommodation, eviction moratoriums, and increases in unemployment benefits and income support (minimum wage) (Boston University, 2023; Rogers & Power, 2020).

At the time of our study, many participants were struggling financially. Although temporary initiatives to mitigate financial risk showed some success (Rogers & Power, 2020), the economic downturns, cost-of-living pressures, and tightening of financial positions currently trending in high-income countries globally suggest that those experiencing social marginalization, and those on the cusp, remain at considerably high risk with respect to housing and health. As supportive initiatives implemented during the pandemic are removed, the disproportionate impacts likely persist, threatening a new wave of young adult housing instability with persistent impact on health (Heerde & Patton, 2020).

### Strengths and Limitations

This study analyzed data collected from a population-based sample, recruited to be state-representative in 2002, and followed prior to and throughout the pandemic, with excellent retention rates. It capitalized on a unique opportunity to examine associations between housing stability and associated vulnerability during the height of pandemic lockdowns in the United States. Findings are from a descriptive cross-sectional analysis. Rates of housing instability are likely subject to underestimation. This study analyzed self-report data; this is considered reliable in studies of young adults (Jolliffe et al., 2003). The findings are generalizable only to the state and cohort sample analyzed.

### Implications for Policy and Research

The impact of social marginalization on health and health inequity, combined with persistent impacts of the pandemic, underscores the need for multisectoral approaches to address the needs of socially marginalized groups during public health crises and beyond. It is important to note that young adults who had not experienced housing instability in this sample also reported health and socioeconomic vulnerabilities arising from the pandemic, but clear inequities exist between those who experience marginalization and those who do not. However, vulnerability to housing instability and susceptibility to health and socioeconomic difficulties do not discriminate, underscoring an urgent need to move the provision of support, aid, and prevention programming upstream to the population level to help all people equally, beginning long before imminent risk for housing instability develops. And unfortunately, research on young adult housing instability and effective strategies to influence the factors that predict it is limited to date. To move upstream, we must first understand potential modifiable causes of housing instability within an individual’s social-ecological context, with a focus on prevention at the earliest possible point in their development. Then, to build the evidence base for strategic policy action, investment in cross-national research, such as the IYDS, would allow for comparisons of different policies to mitigate the risk of housing instability and its associated health risks. For example, a comparison between pandemic responses in the United States and Australia and its influence on young adult health and social outcomes, considering these two countries implemented dramatically different pandemic-related restrictions and lockdowns, could yield key policy implications. Finally, specific research is needed to examine the potential rebound in young adult housing instability post-pandemic and to evaluate potential policies to mitigate this risk.

## References

[bibr1-15248399231217447] Abrams E. M. Szefler S. J. (2020). COVID-19 and the impact of social determinants of health. The Lancet Respiratory Medicine, 8(7), 659–661. 32437646 10.1016/S2213-2600(20)30234-4 PMC7234789

[bibr2-15248399231217447] Amore K. Baker M. Howden-Chapman P. (2011). The ETHOS definition and classification of homelessness: An analysis. European Journal of Homelessness, 5(2), 19–37.

[bibr3-15248399231217447] Boston University. (2023). COVID-19 U.S. State Policies. https://statepolicies.com/policies-by-state/?st=WA

[bibr4-15248399231217447] Gewirtz O’Brien J. R. Auerswald C. English A. Ammerman S. Beharry M. Heerde J. A. Kang M. Naous J. D. P. Santa Maria D. A. E . (2021). Youth experiencing homelessness during the COVID-19 pandemic: Unique needs and practical strategies from international perspectives. Journal of Adolescent Health, 68(2), 236–240. 10.1016/j.jadohealth.2020.11.005 33541600

[bibr5-15248399231217447] Heerde J. A. Pallotta-Chiarolli M. Parolini A. (2020). “I dropped out early”: School disengagement and exclusion among young people experiencing homelessness. In Towl P. Hemphill S. (Eds.), Safe, supportive, and inclusive learning environments for young people in crisis and trauma: Plaiting the rope (pp. 57–68). Routledge. 10.4324/9780429282102

[bibr6-15248399231217447] Heerde J. A. Patton G. C. (2020). The vulnerability of the young homeless. The Lancet Public Health, 5, e302–e303. 10.1016/S2468-2667(20)30121-3 32504582

[bibr7-15248399231217447] Jolliffe D. Farrington D. P. Hawkins J. D. Catalano R. F. Hill K. G. Kosterman R. (2003). Predictive, concurrent, prospective and retrospective validity of self-reported delinquency. Criminal Behaviour and Mental Health, 13(3), 179–197. 14654870 10.1002/cbm.541

[bibr8-15248399231217447] Marmot M. (2022). Recreating society for better health. American Public Health Association.

[bibr9-15248399231217447] McMorris B. Hemphill S. Toumbourou J. Catalano R. Patton G. (2007). Prevalence of substance use and delinquent behavior in adolescents from Victoria, Australia and Washington State, United States. Health Education & Behavior, 34(4), 634–650. 16740513 10.1177/1090198106286272

[bibr10-15248399231217447] Rogers D. Power E. (2020). Housing policy and the COVID-19 pandemic: The importance of housing research during this health emergency. International Journal of Housing Policy, 20(2), 177–183.

[bibr11-15248399231217447] Scales P. C. Benson P. L. Oesterle S. Hill K. G. Hawkins J. D. Pashak T. J. (2016). The dimensions of successful young adult development: A conceptual and measurement framework. Applied Developmental Science, 20(3), 150–174. 30344455 10.1080/10888691.2015.1082429 PMC6176765

[bibr12-15248399231217447] Shaw M. (2004). Housing and public health. Annual Review of Public Health, 25, 397–418. 10.1146/annurev.publhealth.25.101802.123036 15015927

[bibr13-15248399231217447] Temple J. Wood L. Guillot-Wright S. Baumler E. Thiel M. Torres E. (2020). Coronavirus (COVID-19) pandemic questionnaire for youth and young adults. The University of Texas Medical Branch.

[bibr14-15248399231217447] Wang Z. Tang K. (2020). Combating COVID-19: Health equity matters. Nature Medicine, 26(4), 458. 10.1038/s41591-020-0823-6 32284617

